# One‐Stage Arthroplasty or Revision for Seronegative Infections in Hip and Knee

**DOI:** 10.1111/os.12545

**Published:** 2019-12-01

**Authors:** Hao‐yang Wang, Rui Zhang, MD, Ze‐yu Luo, Duan Wang, MD, PhD, Fu‐xing Pei, Xin Tang, Zong‐ke Zhou

**Affiliations:** ^1^ Department of Orthopaedics, West China Hospital/West China School of Medicine Sichuan University Chengdu China; ^2^ Rehabilitation Medicine Center, West China Hospital/West China School of Medicine Sichuan University Chengdu China

**Keywords:** Arthroplasty, One‐stage revision, Seronegative infection

## Abstract

**Objective:**

To assess the safety and effectiveness of one‐stage total joint arthroplasty (TJA) or revision for seronegative infections after total hip arthroplasty (THA) and total knee arthroplasty (TKA).

**Methods:**

This retrospective study included a total of 495 patients who had undergone one‐stage total joint (hip or knee) arthroplasty or revision with a diagnosis of osteoarthritis secondary to sepsis, osteoarthritis or osteonecrosis of the femoral head (ONFH) secondary to internal fixation surgery of the hip joint, and one‐stage revision for prosthesis loosening after THA or TKA from January 2012 to December 2016. Bacterial cultures were taken from all patients (from joint fluid or articular cavity fluid and four to six different parts of soft tissues) during the operation. If the cultures were positive, patients received antibiotic treatment. Microbiology results from surgical samples, clinical evaluations, SF‐12 score (physical component summary [PCS] and mental component summary [MCS]), Harris hip score (HHS) or Hospital for Special Surgery (HSS) score, and patients' satisfaction was recorded at every follow‐up session.

**Results:**

A total of 24 patients had a positive result for bacterial culture (4.85%). The bacterial culture results showed that there were 19 cases (79.16%) of gram‐positive cocci (*Staphylococcus aureus*), 4 cases (16.67%) of gram‐negative bacilli, and 1 case (4.17%) of fungi. For at least 24 months (mean 35 months) follow‐up, no reinfection was discovered. The mean HHS or HSS score improved significantly from 36.29 points preoperatively to 84.21 points postoperatively (*P* < 0.001). The mean PCS score improved from 10.15 preoperatively to 20.34 postoperatively, and the mean MCS from 13.22 preoperatively to 21.76 postoperatively, with significant differences. Most of the patients were satisfied.

**Conclusion:**

One‐stage arthroplasty or revision with exhaustive debridement, adequate dosage, and duration of sensitive antibiotics is safe and effective for patients who have seronegative infection of hip or knee joints.

## Introduction

Total hip arthroplasty (THA) and total knee arthroplasty (TKA) are effective treatments for end‐stage joint diseases and can relieve pain and improve patients' quality of life. Patients are increasingly choosing to undergo such procedures, with the number of patients expected to reach 4 million annually in the United States^1^, ^2^. With the aging population and longer lifespan in China, the number of patients requiring THA or TKA will increase dramatically. However, total joint arthroplasty (TJA) is still associated with many related complications, with periprosthetic joint infection (PJI) being one of the most serious and placing a burden on the healthcare system. Previous studies have shown that the rate of PJI is approximately 0.5%–3% following THA/TKA^3^. Although the incidence of PJI is low, affected patients suffer from severe pain and require revision surgery^4^, which causes enormous social and economic burden.

Revision surgery is an effective treatment for PJI. Recent studies indicate that there are two kinds of treatment for TJA revision: one‐stage revision or two‐stage revision. Although the curative rates of the methods are satisfactory, the two‐stage revision was still regarded as the golden standard for chronic PJI^5^, ^6^. However, some patients who were scheduled for primary or revision surgery had a history of an operation (including internal fixation after fracture and primary THA or TKA) or suppurative arthritis, which may leave bacteria in the joint and cause PJI. Some studies show that previous suppurative arthritis, a history of joint surgery, or internal fixation increase the risk of PJI following TJA. There may be no sign of infection before the arthroplasty surgery, with this phenomenon referred to as seronegative infection or occult infection by some researchers^7^, ^8^. Thus, orthopaedic surgeons may complete the surgery without appropriate laboratory tests being done. If debridement is not conducted intraoperatively for these patients undergoing primary or revision surgery and they do not receive a full course of antibiotics, they will have increased risk of PJI.

For these patients, the choice of one‐stage joint arthroplasty or two‐stage arthroplasty challenging. Many studies show that one‐stage and two‐stage arthroplasty both have good results in treating PJI, but there still no established protocol for treating patients with higher risk of infection after joint arthroplasty or revision surgery.

For this reason, we performed this retrospective study to determine whether: (i) one‐stage arthroplasty or revision can be used to treat patients who a have risk of seronegative infection before the operation; (ii) bacterial culture and debridement are necessary during the surgery; and (iii) the appropriate dosage and duration of antibiotics.

## Methods

The study protocol was approved by the local institutional review board of West China Hospital, Sichuan University. Between January 2011 and December 2016, we identified all patients in our institutional registry: (i) who had been treated for primary THA or TKA following osteoarthritis secondary to sepsis osteoarthritis or osteonecrosis of the femoral head (ONFH) secondary to internal fixation surgery; and (ii) who had undergone one‐stage revision for prosthesis loosening after THA or TKA. Exclusion criteria were as follows: (i) patients treated with primary THA or TKA for primary osteoarthrosis, ONFH caused by alcohol or glucocorticoids, or rheumatoid arthritis; (ii) two‐stage revision for PJI; and (iii) one‐stage revision for periprosthetic fracture. A total of 495 patients were included in this study (436 hips and 59 knees). Of these, 351 were patients who had undertaken THA or TKA (302 hips and 49 knees). Following the diagnostic criteria of the AAOS for PJI, 351 patients were diagnosed with aseptic loosening^9^. Patient demographics (e.g. age, gender, height, weight, BMI, and diagnosis) were recorded. A history of surgery or infection of joints means that bacteria are hidden in tissues and, therefore, patients are at increased risk of PJI after the TJA^7^, ^10^. Bacterial cultures were taken during the operation, and we lengthened the duration of the course of antibiotics and had more frequent follow‐up sessions if there was a positive result. The Harris hip score (HHS) and the SF‐12 score were assessed before the operation and at the end of the follow‐up period. Clinical data of our patients were evaluated retrospectively after receiving approval from the Institutional Review Board of West China Hospital, and written informed consent was obtained from all participants.

### *Surgical Procedures*

#### *Anesthesia and Position*

All patients who underwent one‐stage arthroplasty or revision surgery were given general anesthesia. Patients were placed in the lateral position for hip surgery and the supine position for knee surgery.

#### *Approach, Exposure, and Main Surgery Process*

For the THA/TKA or revision surgery, the conventional surgical procedure was followed. We used the posterolateral approach for hip joint surgery and the medial parapatellar approach for knee surgery, and removed the internal fixation if necessary and cleared away all the scar tissue and hyperplasia tissue with an electrotome. After finishing the debridement, we used hydrogen peroxide and povidone‐iodine to soak the surgical field for 10 min before prosthesis implantation, respectively. For the patients undertaking THA or TKA, we took out the prosthesis, and then an exhaustive debridement was conducted during the operation. Meanwhile, all the granulation tissue, suspicious scarring, and soft tissues between the prosthesis and bone was cleared away. After complete debridement, we also soaked the surgical field with hydrogen peroxide and povidone‐iodine for 10 min, respectively, and washed the surgical field with an auto‐pulse operation rinse before prosthesis implantation.

#### *Postoperative Management*

We used second generation cephalosporins to prevent the infection for approximately 3–5 days until we confirmed the results of the microbial culture. If the bacterial culture was positive, the duration of antibiotic use was prolonged. An antibiotic based on the drug sensitivity results was used for 6 weeks intravenously and then an oral antibiotic was used subsequently for another 6 weeks. For all patients who had a positive bacterial culture, we did the joint cavity paracentesis and took joint fluid to bacterial culture at 1 week and 2 weeks postoperatively and before ceasing the use of antibiotics. Patients were checked monthly while they received antibiotic treatment. After completing the treatment of antibiotics, these patients were followed up every 6 months for a minimum of 24 months. At each follow‐up session at our center, clinical response, erythrocyte sedimentation rate (ESR), and C‐reactive protein (CRP) were examined. With a positive result for bacterial culture, CRP, and ESR, we lengthened the course of antibiotic treatment. The prostheses were all from the same company (Johnson & Johnson), and cementless prostheses were used for the hip and cemented prostheses for the knee.

#### *Intraoperative Microbiology and Histology*

Samples for bacteriological culture were taken at the start of the operation before the administration of antibiotic prophylaxis. If there was no joint fluid, we used saline to irrigate the articular cavity and took some flushing fluid. The samples were injected into aerobic and anaerobic blood culture bottles immediately. Soft tissues from four to six different parts around the joint were taken to conduct pathological examination.

### *Outcome Measurements*

#### *Bacterial Culture, Drug Sensitivity of Antibiotic, and Inflammation Markers*

Bacterial culture: The bacterial culture was assessed using joint fluid and soft tissues during the operation and joint fluid only at the 1 week and 2 weeks after surgery, and at the cessation of antibiotic use by joint cavity paracentesis. If the bacterial culture was positive, it was considered to be infected and the antibiotics would continue to be used.

Drug sensitivity of antibiotics: The results of patients' drug susceptibility results were provided by a microbiology laboratory; the surgeons selected the appropriate antibiotic based on the drug susceptibility results.

Inflammation markers: We chose CRP and ESR as a means of monitoring whether a patient's infection had recurred. Patients were seen monthly while they continued antibiotic treatment and the CRP and ESR were examined at each follow‐up session. If the CRP and ESR had risen to two times the normal value, antibiotic use would be prolonged. If the CRP and ESR showed a continuous decline or stabilized in the normal range, intravenous antibiotics would be changed to oral drugs after 6 weeks; then if the ESR and CRP continued in the normal range, the oral antibiotics were stopped after 6 weeks. After finishing the treatment of antibiotics, the patients were followed every 6 months for a minimum of 24 months.

#### *X‐rays, Function of Knee and Hip, Quality of Life, and Satisfaction*

X‐rays of the surgical joint: For the hip we conducted pelvic positive X‐rays and a positive and oblique X‐rays of the femoral neck; and for the knee, patients underwent positive and lateral X‐rays of the knee. Radiographs of the surgical site were taken for all patients preoperatively and postoperatively, and at the time of last follow‐up to see if there had been prosthesis loosening or bone destruction. The serial radiographs were also evaluated for evidence of component migration, heterotopic ossification, osteolysis, subsidence, and linear polyethylene wear. For the hip, the acetabular component loosening was defined as progressive radiolucent lines of >2 mm around the inserted cup, or migration, or a change in the position of the cup^4^. For the knee, osteolysis was defined as a radiolucent lesion that was a minimum of 5 mm in size with loss of normal trabecular pattern and a sclerotic margin that was not present on the preoperative or immediate postoperative radiograph^5^, ^7^.

The function of the hip and knee: We used the Hospital for Special Surgery (HSS) score for patients who underwent hip surgery and the HSS score for knee surgery. The two scales included the pain and joint function, which were recorded at the time of admission and discharge, and at each follow‐up time point.

Quality of life: The SF‐12 scale included a physical component summary (PCS) and a mental component summary (MCS). The SF‐12 scale was completed by all patients in our study. These three scales were assessed before the operation and at every follow‐up session.

Satisfaction: The satisfaction of patients was measured at the last follow‐up and used the standard of Marsh^11^. The satisfaction was divided into six levels: extremely satisfied, very satisfied, somewhat satisfied, neither satisfied nor dissatisfied, somewhat dissatisfied, and very dissatisfied.

#### *Dislocation, Delayed Wound Healing, and Infection Recurrence*

Postoperative complications, namely, dislocation of the surgery joint, delayed wound healing, infection recurrence, and severe or deadly complications, were recorded.

### *Statistical Analysis*

A two‐sided paired Student *t*‐test was used to analyze preoperative and postoperative continuous variables. Statistical significance was established at *P* < 0.05. The χ^2^‐test was carried out to analyze categorical variables. These data are available in mean values with ranges. Statistical analysis was performed with the use of SPSS statistics software version 21.0 (IBM, Armonk, NY, USA).

## Results

### *Patients*

Of 495 patients, 94 hips had osteoarthritis secondary to sepsis (19%), 40 hips osteoarthritis or ONFH secondary to trauma (8%), 302 hips prothesis loosening after THA (61%), 10 knee osteoarthritis secondary to trauma (2%), and 49 prothesis loosening after TKA (10%). A total of 24 patients had positive bacterial culture (4.85%). There were 2 patients (2.12%) in the cohort of hip osteoarthritis secondary to sepsis, 4 (10.00%) in the cohort of hip osteoarthritis or ONFH secondary to trauma, 14 (4.64%) in the cohort of prothesis loosening after THA, and 4 (8.16%) in the cohort of prothesis loosening after TKA, and no patients were found in the knee osteoarthritis secondary to trauma cohort. The mean time of surgery was approximately 81 minutes (Table 1). All operations were successful and no patients died in our study.

**Table 1:** Main characteristics of the patients included in the study

Variables	Patients (*N* = 495)
Age, mean ± SD	56.9 ± 11.5
Gender (number [%] of patients)	
Male	312 (63.03)
Female	183 (36.97)
Height (cm), mean ± SD	163.14 ± 5.63
Weight (kg), mean ± SD	63.78 ± 3.31
BMI, mean ± SD	24.3 ± 3.2
Diagnosis (number [%] of patients) of patients undertaking one‐stage total joint arthroplasty or revision	
Hip osteoarthritis secondary to sepsis	94 (19)
Hip osteoarthritis or ONFH secondary to trauma	40 (8)
Prothesis loosening after THA	302 (61)
Knee osteoarthritis secondary to sepsis	0 (0)
Knee osteoarthritis secondary to trauma	10 (2)
Prothesis loosening after TKA	49 (10)
Concealed infection (number [%] of patients)	
Hip osteoarthritis secondary to sepsis	2 (2.12)
Hip osteoarthritis or ONFH secondary to trauma	4 (10.00)
Prothesis loosening after THA	14 (4.64)
Knee osteoarthritis secondary to sepsis	0 (0)
Knee osteoarthritis secondary to trauma	0 (0)
Prothesis loosening after TKA	4 (8.16)
Time of surgery(min), mean ± SD	81 ± 19.2
Follow‐up(month), mean ± SD	35.67 ± 4.71

BMI, body mass index; ONFH, osteonecrosis of femoral head; THA, total hip arthroplasty; TKA, total knee arthroplasty.

### *Follow‐Up*

All 24 patients came to the outpatient department for their followup at the first and second weeks after the operation and at the time that they completed their course of antibiotics. The arthrocentesis was done each time they came back to the hospital.

### *Bacterial Culture and Drug Sensitivity Test*

The drug sensitivity test report took an average of 3.1 days. The gram‐positive cocci susceptibility took the shortest time (an average of 2.8 days). The gram‐negative bacteria and fungi took 4 days after the operation. The drug sensitivity results showed that among the 24 cases, there were 2 cases (8%) of multidrug‐resistant bacteria and 22 cases (92%) of non‐multidrug‐resistant bacteria. For all of the drug sensitivity results, the average resistance drugs were 2 species, but the average sensitive antibiotics were 14 species. Even for the two cases of multidrug‐resistant bacteria, almost 20 kinds of antibiotics can be administered.

The results of bacterial culture showed that there were 19 cases (79.16%) of gram‐positive cocci (*Staphylococcus aureus*), 4 cases (16.67%) of gram‐negative bacilli, and 1 case (4.17%) of fungi. We conducted the bacterial culture examination for all 24 patients using joint cavity paracentesis. For 2 of them we found the same bacteria 1 week after the operation, but for the next two tests, at 2 weeks after the operation and at the time of completion of antibiotic treatment, the bacterial culture results were negative. The drug sensitivity results showed that the virulence of these bacteria is not very high and they have a limited destructive capability. After the exhaustive debridement and the use of sensitive antibiotics species, the infection can be controlled effectively.

### *Radiographs*

**Figure 1:**
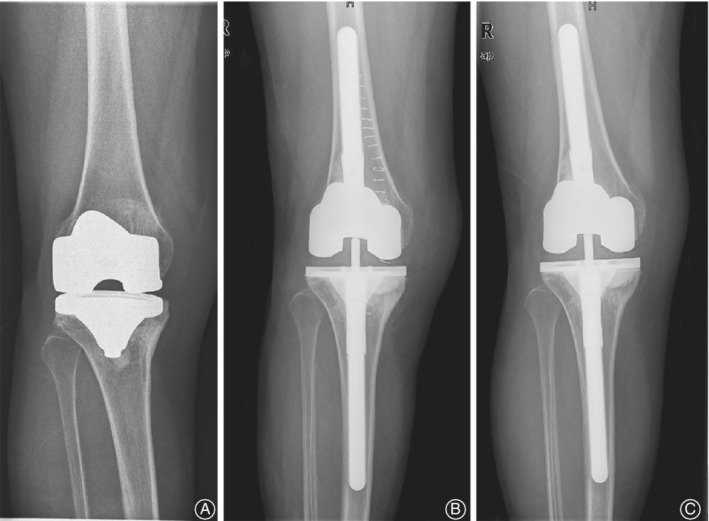
A 64‐year‐old woman, with one‐stage revision of the right knee for prothesis loosening. Bacterial culture of synovial fluid shows the *Staphylococcus epidermidis* infection. (A) 3 years after the primary TKA. (B) 1 day after the revision. (C) 39 months after the revision, no radiolucent lines were found, and no migration, osteolysis, or subsidence were detected. The components were considered to be stable.

**Figure 2:**
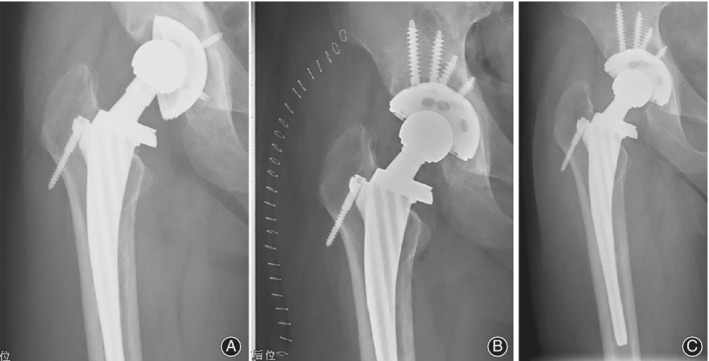
A 48‐year‐old man, with one‐stage revision of the right hip for prothesis loosening and primary THA of the left hip; bacterial culture of synovial fluid from the right hip shows the *Staphylococcus capitis* infection. (A) 8 years after the primary THA. (B) 1 day after the revision of the right hip and the primary THA of the left hip. (C) 35 months after the revision, no radiolucent lines were found. No migration, osteolysis, or subsidence were detected. The components were considered to be stable.

**Figure 3:**
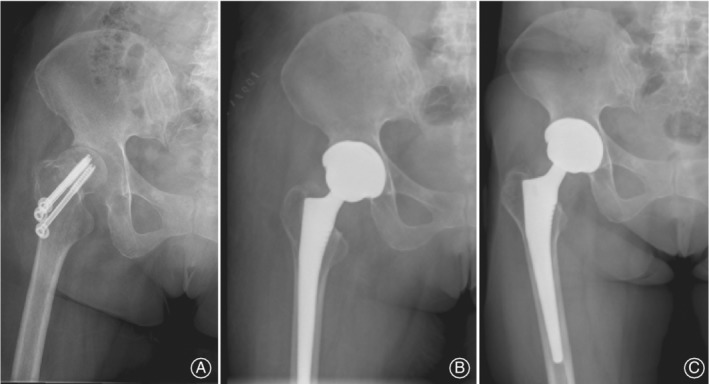
A 42‐year‐old woman underwent one‐stage arthroplasty of the right hip for failure of the femoral neck fracture at the right side. Bacterial culture of synovial fluid from the right hip shows the *Staphylococcus epidermidis* infection. (A) 2 years after the internal fixation of the femoral neck. (B) 1 day after primary THA of the right hip. (C) 29 months after primary THA of the right hip. No radiolucent lines were found. No migration, osteolysis, or subsidence were detected. The components were considered to be stable.

None of the patients were lost to follow up, with a mean follow up of 35 months (range 27–50). All patients were cured and no radiological signs of loosening and infection were observed (Fig. 1 Fig. 2 and Fig. 3).

### *Harris Hip Score and Hospital for Special Surgery Score*

The mean HHS and HSS score before the operation was 36.29. At the end of the follow up, the mean joint functional score was 84.21 (*P* = 0.015); the difference was statistically significant.

### *Quality of Life and Satisfaction*

The mean PCS of SF‐12 rose from 10.17 before the operation to 20.25 at the end of follow up, and the mean MCS rose from 13.13 to 24.25; the difference was statistically significant.

The satisfaction of patients was measured using the standard of Marsh:^11^ 7 (29.17%) patients felt extremely satisfied, 12 (50%) patients felt very satisfied, 4 (16.7%) patients felt somewhat satisfied and 1 felt neither satisfied nor dissatisfied. Patients who underwent the hip arthroplasty or revision surgery felt more satisfied than those who underwent knee arthroplasty.

### *Complications*

There was 1 patient who suffered a dislocation of the hip joint 3 weeks after the operation and had a successful manual reduction. There were no severe or deadly complications in this study. There was no recurrence of infection in the 24 patients who had a positive etiology result.

## Discussion

One‐stage arthroplasty or revision can be used to treat patients at risk of seronegative infections. PJI is a problem that patients and surgeons cannot avoid. Infections of the urinary tract, the respiratory tract, the digestive tract, and the oral cavity may all lead to PJI. It is pointed out in the published literature that being male, smoking, obesity, glucocorticoid use, and joint surgery history are risk factors of PJI^12^, ^13^, ^14^, ^15^. Treatment for chronic PJI with one‐stage or two‐stage revision is still controversial.

The current gold standard for the treatment of chronic PJI is the two‐stage revision, removing the original prosthesis and implanting the antibiotic cement spacer after thorough debridement; 4–6 weeks intravenous and 4–6 weeks oral antibiotic treatment follows, and a new prosthesis is implanted after the infection is controlled^3^, ^16^. Drexler *et al*. treated patients with chronic PJI with two‐stage revision after TKA; the success rate of the two‐stage revision surgery was up to 85.4%^17^. Hoell's study found that the two‐stage revision for chronic PJI of TKA had a success rate of 93.2% for 4 years of follow up^16^. The two‐stage revision has a high success rate for TJI. Even if the infection has not been controlled after the first stage operation, doctors could do the debridement and implant another antibiotic spacer until the infection is controlled^3^, ^5^, ^18^, ^19^. However, there are still some disadvantages of two‐stage revision. For example, patients who choose the two‐stage revision must be able to withstand repeated surgery and anesthesia. Some people may not tolerate repeated surgery and anesthesia and are unable to complete treatment. Lee *et al*. follow up some elderly patients who were unable to complete the two‐stage revision and were obliged to use antibiotic cement spacers as the ultimate treatment after the first step of the two‐stage revision for chronic peripheral infection of THA. The results showed that although the joint function for those who used the prefabricated temporary antibiotic cement spacer as the ultimate treatment was not as not good as for patients who underwent the two‐stage revision, the differences had no statistical significance^20^. Ilchmann *et al*. used one‐stage revision treatment for 38 patients with chronic PJI after THA and these patients were followed up for 2–15 years. The results showed that most of these patients had good clinical outcomes. There were 4 patients who suffered aseptic loosening of the prosthesis, but infection did not recur^21^. The results of a meta‐analysis showed that there was no statistically significant difference in the rate of infection recurrence between one‐stage and two‐stage revision after PJI following TJA^6^, ^22^, ^23^, ^24^. Debate continues about whether to choose one‐stage or two‐stage revision to treat PJI, and this question has attracted increasing interest by joint surgeons. To further explore this issue, Strange *et al*. launched a multistate, multicenter, prospective, randomized controlled study^25^.

Preoperative examination is the best way to diagnose PJI. Inflammation markers like ESR and CRP are frequently used in the clinic. According to the diagnosis of PJI of the hip and knee of AAOS, patients with a CRP over 10 mg/L have a great chance of PJI^26^, ^27^, ^28^. However, patients with low inflammation markers still have the possibility of PJI. McArthur discovered that approximately 4% of patients with PJI have a normal inflammation marker^29^. According to his advice and the guidelines of the AAOS, in patients with suspected infection, joint punctures should be performed to extract synovial fluid, followed by cell counting and bacterial culture to screen for infection. However, in the clinical work, some patients had little synovial fluid, which could not be removed, even with ultrasound guidance. Moreover, preoperative punctures may take bacteria follicle in joint cavity. If disinfection is not strictly; this will increase the PJI rate. In short, diagnosis of seronegative infections is very hard. The clinician needs to focus on how best to treat seronegative infections during and after the operation as well as ensuring patients' safety. The results of our study show that although the seronegative infections are dormant and unpredictable, if the debridement during the operation is exhaustive and the use of antibiotics is timely, the one stage arthroplasty or revision surgery can have a good result.

### *Bacterial Culture and Debridement are Necessary*

Hip and knee osteoarthritis secondary to sepsis, hip osteoarthritis, and ONFH secondary to trauma are common hip and knee secondary diseases that need to be treated with joint replacement. The number of patients undergoing revision due to loosening and PJI after primary hip and knee arthroplasty is increasing day by day. In the United States, approximately 17% of THA patients need to undergo revision surgery. In the last ten years, total joint replacement has developed rapidly in China, and there will continue to be a large number of revision cases. Scars near the surgical site, bone deformities, and internal fixation shields make joint replacement or revision surgery more difficult. In addition, hip and knee replacement in patients with previous surgery or infection are associated with a greater risk of infection and complications^10^, ^30^. Schwarzkopf *et al*. found that THA after the failure of hip fracture internal fixation had greater postoperative infection incidence than primary THA^7^, ^31^, ^32^, ^33^. Gallo *et al*. indicated that when a prosthesis or implant is implanted in the human body, bacteria may colonize to the surface of the prosthesis by surface adhesion and hematogenous migration, with nearly one‐third of bacteria transmitted by blood circulation^34^. Most of these patients have normal laboratory tests before the operation. We must pay more attention to them both during and after the surgery.

Under the control of the immune system, colonized bacteria may be in a lag phase for a long time and coexist with the patient's body. Reoperation may break the balance between colonization bacteria and the host, and release bacteria which had been trapped in the scar tissue or the surface of the plant. This may be the cause of seronegative infections. Therefore, we must do the bacterial culture during the surgery so as not to leave out a “carrier.”

One‐stage arthroplasty is an effective method for the treatment of secondary osteoarthritis post‐infection or failure of surgery after trauma; one‐stage revision treatment for TJI after TJA also provides satisfactory results. In addition, the one‐stage arthroplasty or revision can effectively relieve pain, reconstruct the joint function, and improve the quality of life of patients. In addition, this method can reduce the number of surgeries required. To our knowledge, most of the previous findings suggest that single‐stage and two‐stage revision can both achieve satisfactory clinical outcomes after prosthesis infection (Table 2)^5^, ^21^, ^35^, ^36^, ^37^, ^38^. In our study, these 24 patients also had good outcomes in terms of their joint function and daily life; the one‐stage TJA or revision surgery for patients who had seronegative infections can give them a better life and did not increase the rate of infection. In addition, a relevant systematic review and meta‐analysis also indicated that signal‐stage revision is a reliable procedure for prosthesis infection^2^, ^5^, ^22^, ^24^. What can we do to make sure that patients with seronegative infection of the hip or knee joint have a good treatment result? Based on the experience of former studies, during the operation, exhaustive debridement should be done to remove all necrotic and infectious tissues, implants, and cement. In addition, after the debridement, we used hydrogen peroxide and iodophor to soak the surgical site for 10 min and removed by an auto‐pulse operation rinse matching, which is important to eradicate infection^39^, ^40^. During the surgical procedure, we strictly followed this protocol and no reinfection occurred postoperatively; exhaustive debridement is a very useful and effective strategy to treat patients with seronegative infection of hip or knee joints.

**Table 2:** Main characteristics of studies of one‐stage versus two‐stage revision and one‐stage only

Authors Year of study	Type of surgery	Duration of antibiotics use	Rate of infection control	Mean follow‐up
Knee				
One‐stage revision versus two‐stage revision				
Lecuire and Collodel^41^ 1999	One‐stage	Intravenous	One‐stage	79.2 months
	(*n* = 16)	21 days	93.80%	
	Two‐stage	Oral	Two‐stage	
	(*n* = 41)	6 months	97.60%	
Oussedik and Dodd^42^ 2010	One‐stage	Intravenous	One‐stage	81.6 months
	(*n* = 11)	5 days	100.00%	
	Two‐stage	Oral	Two‐stage	
	(*n* = 39)	6 weeks	94.90%	
Klouche and Leonard^38^ 2012	One‐stage	Intravenous	One‐stage	24 months
	(*n* = 38)	6 weeks	100.00%	
	Two‐stage	Oral	Two‐stage	
	(*n* = 46)	6 weeks	97.80%	
Choi and Kwon^37^ 2013	One‐stage	Intravenous	One‐stage	61 months
	(*n* = 17)	6 weeks	82.00%	
	Two‐stage	Oral	Two‐stage	
	(*n* = 44)	—	71.50%	
Wolf and Clar^43^ 2014	One‐stage	6 weeks in total or 2 weeks after drug sensitivity test	One‐stage	24 months
	(*n* = 37)		56.80%	
	Two‐stage		Two‐stage	
	(*n* = 55)		94.50%	
Li and Hou^44^ 2015	One‐stage	Intravenous and oral	One‐stage	103.2 months
	(*n* = 6)	for 6–12 weeks	100.00%	
	Two‐stage		Two‐stage	
	(*n* = 4)		100.00%	
One‐stage revision				
Wroblewski^45^ 1986	One‐stage	Intravenous	One‐stage	38 months
	(*n* = 102)	2 days	91.00%	
		Oral		
		6 weeks		
Raut and Siney^46^ 1994	One‐stage	Intravenous	One‐stage	88 months
	(*n* = 57)	from operation to sensitivity results	86.00%	
		Oral		
		6 weeks to 3 months		
Raut and Siney^47^ 1995	One‐stage	Intravenous	One‐stage	93 months
	(*n* = 183)	4 weeks (2 patients)	84.20%	
		Oral		
		6 weeks to 3 months (146 patients)		
Rudelli and Uip^48^ 2008	One‐stage	Intravenous	One‐stage	103 months
	(*n* = 32)	at least 4 weeks	93.80%	
		Oral		
		maintained to 6 months postoperation		
Yoo and Kwon^49^ 2009	One‐stage	Intravenous	One‐stage	86.4 months
	(*n* = 12)	4.9 weeks for all	91.67%	
		Oral		
		6 weeks for 7 of 12		
Singer and Merz^50^ 2012	One‐stage	Intravenous	One‐stage	36 months
	(*n* = 63)	2 weeks	95.00%	
		Oral		
		4 weeks		
Bori and Mahamud^36^ 2014	One‐stage	Intravenous	One‐stage	44.6 months
	(*n* = 24)	10 days	95.80%	
		Oral		
		50.1 days		
Zeller and Lhotellier^51^ 2014	One‐stage	Intravenous	One‐stage	41.6 months
	(*n* = 157)	4 to 6 weeks	94.90%	
		Oral		
		6 to 8 weeks		
Knee				
One‐stage revision versus two‐stage revision				
Scott and Stockley^52^ 1993	One‐stage	Not concern	One‐stage	Not concern
	(*n* = 10)		70.00%	
	Two‐stage		Two‐stage	
	(*n* = 7)		100.00%	
Buechel and Femino^53^ 2004	One‐stage	Intravenous	One‐stage	122.4 months
	(*n* = 22)	4–6 weeks	90.90%	
		Oral		
		6–12 months		
Laffer and Graber^54^ 2006	One‐stage	67.6% use for more than 6 months, 32.3% use less than 6 months	One‐stage	28 months
	(*n* = 21)		100.00%	
	Two‐stage		Two‐stage	
	(*n* = 13)		84.60%	
Prasad and Paringe^55^ 2014	One‐stage	Intravenous	One‐stage	60 months
	(*n* = 26)	5 days	88.00%	
	Two‐stage	Oral	Two‐stage	
	(*n* = 34)	6 weeks	94.00%	
Haddad and Sukeik^56^ 2015	One‐stage (*n* = 28)	1 to 6 weeks intravenous	One‐stage 100.00%	78 months
	Two‐stage (*n* = 74)	5 days intravenous continue for 6 weeks Intravenous or oral	Two‐stage 93.00%	
Massin and Delory^57^ 2016	One‐stage	total 6 weeks	One‐stage	One‐stage
	(*n* = 108)		79.00%	44 months
	Two‐stage		Two‐stage	Two‐stage
	(*n* = 177)		69.00%	55 months
One‐stage revision				
Singer and Merz^50^ 2012	One‐stage	Intravenous	One‐stage	36 months
	(*n* = 63)	2 weeks	95.00%	
		Oral		
		4 weeks		
Tibrewal and Malagelada^58^ 2014	One‐stage	Intravenous	One‐stage	126 months
	(*n* = 50)	2 weeks	98.00%	
		Oral		
		3 months		
Labruyere and Zeller^59^ 2015	One‐stage	Intravenous	One‐stage	60 months
	(*n* = 9)	6 weeks	100.00%	
		Oral		
		6 weeks		
Zahar and Kendoff^60^ 2016	One‐stage	Intravenous	One‐stage	120 months
	(*n* = 70)	14.2 days	93.00%	
		Oral		
		none		

Ensuring adequate dosage and duration of sensitive antibiotics is important. Because the results for intraoperative cultures and antibiotic susceptibility testing would be available approximately 3 days after the surgery, antibiotics were chosen according to our hospital protocol before we obtained the culture results and then antibiotics were adjusted on the basis of subsequent intraoperative cultures and antibiotic susceptibility testing. We checked studies on one‐stage or two‐stage total joint revision published in recent years. The results showed that both methods have satisfactory results with prolonged antibiotics use. Compared with multiple resistant bacteria, the virulence of the bacteria found in our study is relatively weak and has a low rate of antibiotics resistance. Our data show that in patients with seronegative infections around the joint, 92% of the bacteria obtained from synovial fluid or articular cavity were non‐multidrug‐resistant bacteria; even with multidrug‐resistant bacteria, antimicrobial susceptibility results showed that at least 10 antimicrobials were effective against the bacterium. For the antibiotics regimen, we recommend 6 weeks intravenous followed by 6 weeks of oral antibiotics, and the result is good (Table 3). Ensuring adequate dosage and duration of sensitive antibiotics for seronegative infection is necessary.

**Table 3:** The information of laboratory examination and clinical outcomes before and after operation

Number	Surgery	ESR	CRP	WBC	Time for drug sensitivity (Days)	Culture	Number of antibiotics	Multiple resistant bacteria	Antibiotics	Harri hip score	Harri hip score	PCS	PCS	Follow up (months)
	site	(mm/h)	(mg/L)	(10^9^/L)		Introp.	Sensitive		IV	Or	Or	MCS	MCS	
				NEUT		1 week	Insensitivity		Oral	HSS score	HSS score	Preop.	Postop.	
				(%)		2 weeks				Preop.	Postop.			
						Finish drug use								
1	Hip	19	5.66	6.14	3	*Staphylococcus epidermidis*	17	No	Va + Ce	36	90	9	20	40
				56.3		No	0		Ri			14	23	
						No								
2	Hip	30	7.19	7.45	3	*Staphylococcus epidermidis*	19	No	Va + Ce	32	89	11	19	36
				66.1		No	0		Ri			13	22	
						No								
3	Hip	17	4.06	7.02	3	*Staphylococcus aureus*	21	Yes	Ce + Su	34	87	11	19	39
				74.3		Yes	6		Ri			15	26	
						No								
4	Hip	12	6.51	8.13	3	*Staphylococcus capitis*	12	No	Va + Le	32	92	8	18	38
				58.4		No	0		Ri			13	25	
						No								
5	Hip	16	2.31	6.42	2	*Staphylococcus epidermidis*	14	No	Va + Le	28	90	9	22	34
				58.4		No	2		Ri			10	20	
						No								
6	Hip	31	3.35	3.23	3	*Staphylococcus capitis*	16	No	Va + Ce	21	85	10	22	29
				65.2		No	4		Ri			11	22	
						No								
7	Hip	27	5.3	8.21	4	*Sphingomonas paucimobilis*	10	No	Ce + Le	36	84	10	20	36
				59.5		No	2		Le			11	22	
						No								
8	Hip	44	6.85	5.25	3	*Staphylococcus epidermidis*	12	No	Va + Ce	24	89	7	17	29
				56.9		No	2		Ri			13	24	
						No								
9	Hip	63	6.3	7.1	3	*Staphylococcus aureus*	14	No	Ce + Su	31	92	7	19	36
				59.4		No	2		Ri			14	29	
						No								
10	Hip	24	3.83	5.53	4	*Sphingomonas paucimobilis*	15	No	Le	30	96	14	24	35
				67.8		No	2		Le			16	24	
						No								
11	Hip	38	6.27	6.6	2	*Staphylococcus capitis*	10	No	Va + Ce	29	91	6	20	35
				59.2		No	0		Ri			13	25	
						No								
12	Hip	11	1	5.7	4	Klebsiella oxytoca	23	No	Le + Ce + Su	10	89	9	22	29
				56.7		No	5					12	27	
						No								
13	Hip	21	1	4.22	2	*Staphylococcus capitis*	13	No	Va + Le	44	77	10	21	29
				46.6		No	0		Ri			12	28	
						No								
14	Hip	18	4.32	5.32	4	Klebsiella oxytoca	16	No	Le + Ce + Su	32	79	15	19	30
				48.9		No	2		Le			15	27	
						No								
15	Hip	17	5.12	6.39	3	*Staphylococcus epidermidis*	11	No	Va + Ce	56	82	9	23	38
				55.2		No	2		Ri			14	27	
						No								
16	Hip	26	6.01	6.67	3	Staphylococcus saprophyticus	16	No	Va	34	87	13	24	37
				52.4		Yes	3		Ri			13	26	
						No								
17	Hip	14	2.23	7.01	3	*Staphylococcus epidermidis*	11	No	Va + Le	47	69	11	15	49
				68.5		No	2		Ri			13	23	
						No								
18	Hip	31	4.87	8.13	2	*Staphylococcus capitis*	13	No	Va	41	76	12	18	40
				62.5		No	2		Ri			13	21	
						No								
19	Hip	22	6.65	7.49	3	*Staphylococcus epidermidis*	13	No	Va	39	79	11	19	44
				49.8		No	2		Ri			16	20	
						No								
20	Hip	16	1.05	4.66	3	Staphylococcus saprophyticus	8	No	Va + Le	38	82	9	21	50
				50.2		No	2		Ri			17	29	
						No								
21	Knee	14	7.3	8.75	2	*Staphylococcus capitis*	15	No	Va	48	74	13	23	39
				63.12		No	2		Ri			11	22	
						No								
22	Knee	15	7.85	8.82	4	Candida	5	No	Fl	51	85	9	24	38
				65.4		No	1		Fl			12	21	
						No								
23	Knee	32	6.48	8.93	4	*Staphylococcus epidermidis*	15	No	Va + Ce	50	88	10	20	28
				66.9		No	2		Ri			14	28	
						No								
24	Knee	29	7.98	8.52	3	*Staphylococcus epidermidis*	19	Yes	Va + Le	48	69	11	17	27
				70.1		No	5		Ri			10	21	
						No								

ESR, erythrocyte sedimentation rate; CRP, c‐reactive protein; WBC, white blood cell; NEUT, neutrophil count.

### *Limitations of the Study*

The current study had some limitations. First, this was a retrospective study. Second, the sample size was small and the follow‐up period was short. However, this is just a preliminary report. We will continue this study using a larger sample size and a longer follow‐up time; this will make our study more meaningful.

### Conclusion

For patients with seronegative infection who need to undergo arthroplasty or revision surgery, one‐stage arthroplasty or revision may be associated with a greater risk of recurrence of infection after surgery. However, one‐stage joint replacement or revision surgery can also bring about benefits: patients may need to undergo less surgery and anesthesia, thus saving on medical costs. Such operations require joint surgeons to have higher surgical skills and the ability to discriminate abnormal tissues during surgery. Andrew *et al*. suggests that surgeons' experience, surgical techniques, the type of infection, and hospital infrastructure and conditions may all affect the choice of surgical options for treatment of patients with PJI^41^. Complete debridement is the most important procedure for treating seronegative infection when undergoing one‐stage TJA or revision; adequate dosage and duration of sensitive antibiotics is necessary. Although the results of our study and related research have shown that one‐stage TJA and revision surgery are safe and effective for patients who have seronegative infection of hip or knee joints, the choice to undergo this treatment in China still needs to be made carefully because of the increasingly tense doctor–patient relationship, and we need a larger sample and higher quality clinical study to confirm this opinion.
